# Vascularity Assessment of Parathyroid Glands Using Low-Dose ICG and Probe-Based Fluorescence Detection

**DOI:** 10.1245/s10434-026-19250-8

**Published:** 2026-02-20

**Authors:** Parker A. Willmon, Giju Thomas, Colleen M. Kiernan, Naira Baregamian, Carmen C. Solórzano, Anita Mahadevan-Jansen

**Affiliations:** 1 Department of Biomedical Engineering, Vanderbilt University, Nashville, TN USA; 2 Vanderbilt Biophotonics Center, Vanderbilt University, Nashville, TN USA; 3 Department of Surgery, Vanderbilt University Medical Center, Nashville, TN USA; 4 Division of Surgical Oncology and Endocrine Surgery, Vanderbilt University Medical Center, Nashville, TN USA

**Keywords:** Near infrared autofluorescence, NIRAF, Indocyanine green, Low dose, Parathyroid glands, Angiography

## Abstract

**Background:**

Indocyanine green (ICG) angiography is used to assess parathyroid gland perfusion after thyroidectomy to reduce rates of hypocalcemia. This study was conducted to evaluate the efficacy of probe-based fluorescence detection of parathyroid glands with ICG for quantitative perfusion assessment.

**Methods:**

Patients undergoing endocrine neck surgery were recruited for a prospective study. Parathyroid glands were identified intraoperatively using near infrared autofluorescence (NIRAF) with a probe-based system. A 0.25 mg dose of ICG was administered to patients before surgical closure, and the fluorescence from the parathyroid gland was measured 3 min after injection and compared with pre-ICG NIRAF. Images from ICG angiography were collected and scored by three surgeons to analyze inter-rater variability. Probe-measured ICG ratios were compared with ICG image scores to determine the utility of probe-based ICG perfusion quantification in parathyroid glands.

**Results:**

This study analyzed 25 patients. Analysis of ICG image scores showed moderate agreement (*κ* = 0.48–0.58). However, there was significant disagreement when glands with poor blood flow, validating the need for a quantitative approach in ICG angiography. A metric of probe-measured fluorescence change after ICG was developed to classify ligated and untouched glands from parathyroidectomies. A ≤ 1.0-fold increase in fluorescence corresponded with devascularized glands, whereas a ≥ 4.5.-fold increase identified well-vascularized glands. Fluorescence between $1.0$- and $4.5$-fold identified glands with poor flow.

**Conclusion:**

Scoring of ICG angiography is highly subjective and varies significantly among surgeons. However, probe-based fluorescence measurements after low-dose ICG administration can quantitatively predict the vascularity status of parathyroid glands.

**Supplementary Information:**

The online version contains supplementary material available at 10.1245/s10434-026-19250-8.

Annually, an estimated 90,000–150,000 thyroidectomies to remove diseased tissue are performed in the United States.^1,2^ These procedures can cause hypoparathyroidism if the parathyroid glands are damaged intraoperatively, potentially leading to severe complications.^3^ Parathyroid glands (PGs) regulate calcium levels by secreting parathyroid hormone (PTH). When these glands are damaged, PTH production decreases, causing hypocalcemia. Approximately 19–38% of patients who undergo total thyroidectomy experience transient hypocalcemia, and up to 12% may experience permanent hypocalcemia.^4–6^ Thyroid cancer patients who require central neck dissection also show a greater risk of hypoparathyroidism than patients who do not.^7,8^ The symptoms of PTH underproduction can be severe, including muscle spasms, tetany, and seizures.^9^ Patients are typically prescribed calcium supplements to manage these symptoms, but long-term ingestion of calcium supplements has been linked to cardiovascular diseases and kidney stones.^10^ Thus, there is a need to preserve PGs during thyroidectomy using surgical guidance technologies.

Accidental, partial, or total removal of PGs is a major cause of hypoparathyroidism. Misidentification of PGs can occur due to their size, similarity to surrounding tissues, and location variability.^11^ Studies have demonstrated that PG detection using near infrared autofluorescence (NIRAF) is an effective tool for guiding endocrine surgery.^12–15^ To achieve this, imaging- and probe-based systems have been developed and tested for exciting and collecting NIRAF from both the thyroid and PGs. Parathyroid glands have been shown to fluoresce at least 20% brighter than a patient’s thyroid with 93–100% accuracy.^13,14^

Because PGs share their vasculature with the thyroid, damage to the blood vessels during thyroidectomy can also compromise PG viability^4^. When devascularization is recognized, PG function can be saved through autotransplantation into a muscle pocket^4,16^. However, variability in autotransplantation success rates^4,16–19^ prompts surgeons to seek confirmation that glands are truly devascularized.

Indocyanine green (ICG) angiography has been used to evaluate gland viability.^20,21^ The ICG is injected intravenously in doses of 2.5–10 mg,^20^ causing regions of robust blood flow to fluoresce brightly, whereas poorly perfused regions remain dark when imaged with a near infrared camera. Each gland is then subjectively scored using a three-point scale based on the visually observed fluorescence as follows: 0 (dark glands), 1 (dim or heterogeneous fluorescence), and 2 (bright fluorescence),^22–24^ as shown in Fig. 1.

**Fig. 1:**
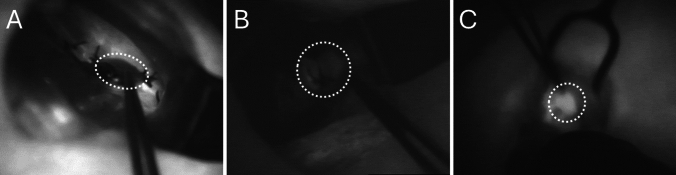
Examples of parathyroid glands from this study with ICG scores of 0, 1, and 2, respectively. The relevant glands are circled

Although this subjective technique provides an approach for evaluating PG perfusion, the utility of ICG angiography during thyroidectomy is inconclusive. Some studies conclude that a single well-scored gland is sufficient to maintain healthy PG function,^23,24^ whereas others recommend two well-perfused glands^25,26^ or suggest a required average or total fluorescence score,^27,28^ and some conclude that there is no benefit from using angiography.^29^ The disagreement likely arises from the unstandardized and subjective ways of assessing perfusion with ICG. Furthermore, the fluorescence spectra of ICG overlaps with the NIRAF of PGs,^12,30^ limiting NIRAF use for PG identification after ICG injection. Therefore, a need exists to develop an objective standardized metric of PG vascularization to ensure consistent results across patients, surgeons, and institutions.

In this study, we hypothesized that probe-based near infrared fluorescence detection can be used to measure PG perfusion quantitatively while simultaneously reducing the necessary ICG dosage. We demonstrated a method to evaluate PG perfusion with a 0.25 mg (0.1 ml) dose of ICG 3 min after injection during thyroidectomies and parathyroidectomies. The numeric value provided by the probe system is stratified to distinguish between devascularized, poorly vascularized, and well-vascularized glands. This stratification offers a quantitative metric of ICG in the blood without the surgeon’s subjective interpretation when using current imaging methods.

## Materials and Methods

### Patient Recruitment

This study was approved by the Vanderbilt University Medical Center Institutional Review Board (IRB no. 141648), and written informed consent was obtained from each patient. Adult patients with thyroid or parathyroid disease were recruited prospectively. If a patient had contraindications for ICG, such as iodide allergies, liver disease, or secondary/tertiary hyperparathyroidism, they were excluded from the study.

The study recruited 25 patients undergoing primary parathyroidectomy (*n* = 9; $\mathrm{PG},27$), total thyroidectomy (*n* = 8; PG,$16$), or lobectomy (*n* = 8; PG, 16), and 59 PGs were assessed. All identified glands were interrogated with the probe.

### Experimental Protocol

This study included three surgeons (C.K., N.B., C.S.), who had been practicing for 6, 11, and 23 years, respectively. All glands were confirmed using the PTeye (Medtronic, Jacksonville, FL, USA). This system takes five autofluorescence measurements from the thyroid with a sterile handheld probe to establish a baseline. Potential PGs are then confirmed by creating a ratio between PG autofluorescence and the thyroid baseline. Autofluorescence detection ratios of 1.2 or greater are audibly confirmed to indicate PG tissue.

During parathyroidectomy, the diseased PGs were ligated, and at least one additional gland was identified by the surgeon. After ligation of the PG, a 0.1 ml dose of 2.5 mg/ml ICG solution (0.25 mg) was administered intravenously, followed by a saline flush. This dosage was chosen because it is the smallest possible dose that can be prepared with the equipment available in the operating suite. Next, 3 min after the injection of ICG, a fluorescence ratio was measured a second time on each PG with the probe. This 3 min delay was used to allow the blood to become well-mixed. Liver clearance was not an issue due to the slow clearance rate of low-dose ICG.^30–32^ This same protocol was followed for thyroidectomies, with ICG injected after thyroid removal.

Images then were captured with the pde-neo II (Hamamatsu Photonics, Bridgewater, NJ, USA) with the room lights and headlamp turned off. The illumination intensity of the imaging system was set to maximum, and the contrast was set to 70%. Images were collected from roughly 30 cm away. In 15 of the 25 cases, an additional 6.25 mg of ICG was injected to enhance the signal for imaging. A larger dose was necessary in these cases because the 0.25 mg dose did not produce a visually detectable increase in tissue fluorescence with the camera. The remaining 10 patients received only 0.25 mg of ICG. No fluorescence ratios were collected with the probe system after the second high-dose injection. The total time for all fluorescence measurements was less than 7 min, and the ICG measurements did not change patient care.

### Data Analysis

Probe-detected fluorescence ratios from each PG were recorded before and after ICG injection, and a “vascularity index” (the gland’s degree of ICG uptake) was calculated by normalizing the post-ICG fluorescence to the corresponding gland’s pre-ICG autofluorescence. The three surgeons (C. K., N. B., C. S.) participating in the study independently scored the acquired ICG fluorescence images of each PG using different computers. Cases were not presented chronologically, and all the surgeons scored the ICG images in the same order. Dark glands were scored as 0, whereas PGs with moderate ICG fluorescence were scored as 1, and those with strong fluorescence were scored as 2. The kappa statistic was calculated to assess the agreement in image scores between each pair of surgeons, with a $\kappa$ of 0 representing chance agreement and a $\kappa$ of 1 representing perfect agreement.^33^ Differences in probe-based vascularity indices and ICG image scores from each surgeon then were assessed by performing a non-parametric ANOVA.^34^ A Dunn’s non-parametric post hoc test with a multiple comparisons adjustment then was applied to each surgeon to identify ICG image scores that differed significantly.^34^ All statistical analyses were performed using MATLAB (MathWorks Inc., Portola Valley, CA, USA). The vascularity index then was compared between the ligated and unligated PGs from the parathyroidectomy cases to establish cutoffs for ICG scores of 0 and 2. Finally, this metric was then applied to PGs from thyroidectomy cases to compare the results with median ICG image scores.

## Results

The study evaluated 59 PGs from 25 patients. Demographics of the recruited patients are shown in Table 1. For 32 PGs, the same ICG image score was received from all three surgeons (Table 2). Of these, 14 glands received a consensus image score of 2, and 14 glands received a consensus score of 0. Only four glands received a score of 1 by all the surgeons. The remaining 27 glands were not scored consistently by the three surgeons, and two of these glands (nos. 37 and 53) were scored differently by each surgeon. Table 2 also shows the kappa statistics (the level of agreement) between pairs of surgeons based on their ICG image scores. The kappa statistics ranged from 0.48 to 0.56, indicating moderate agreement.

**Table 1:** Patient Characteristics and indications for sugery

Sex, n (%)	
Female	17 (68%)
Male	8 (32%)
Race/ethnicity, n(%)	
White	19 (76%)
Non-white	6 (24%)
Age, mean ± SD (years)	53.7±15.5
Indications for sugery, n (%)	
Papillary carcinoma	3 (12%)
Other thyroid cancer	2 (8%)
Goiter	5 (20%)
Nodule	4 (16%)
Graves' disease	2 (8%)
Hyperparathyroidism	9 (36%)

**Table 2:** Indocyanine green (ICG) scores for all parathyroid glands with surgeon disagreement and kappa (κ) statistics

	3	4	5	7	10	12	13	14	15	18	19	20	21	26	29	32	35	37	38	41	42	49	51	53	55	56	57
Surgeon 1	1	2	2	2	1	2	1	1	2	1	2	1	1	1	1	1	1	**1**	1	0	2	2	1	**1**	2	1	1
Surgeon 2	2	2	2	1	0	2	2	1	1	2	2	1	2	2	0	1	0	**2**	2	1	2	2	2	**2**	2	2	0
Surgeon 3	1	1	1	1	0	1	2	0	1	1	1	0	1	1	1	0	0	**0**	1	0	1	1	1	**0**	1	2	1

κ Statistics	Surgeon 1	Surgeon 2	Surgeon 3
Surgeon 1	–	0.56 (0.39–0.73)	0.55 (0.37–0.72)
Surgeon 2	0.56 (0.39–0.73)	–	0.48 (0.30–0.66)
Surgeon 3	0.55 (0.37–0.72)	0.48 (0.30–0.66)	–

ICG image scores with no consensus are bolded

**Fig. 2:**
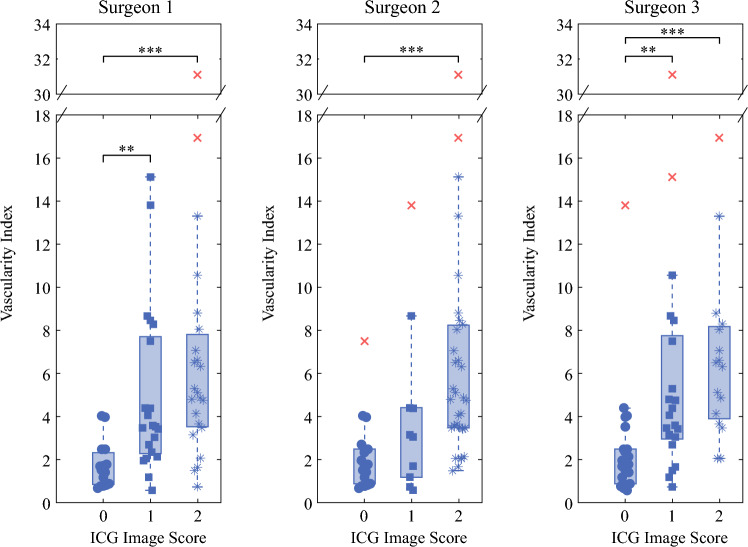
Distribution of PTeye vascularity indices compared to the three-minute ICG image scores for Surgeon 1, Surgeon 2, and Surgeon 3 at a 0.1 ml dose. Data points designated as outliers by the Kruskal-Wallis test are marked with an X. (**: *p* < 0.01,***:*p*<0.001)

Figure 2 shows the probe-based vascularity indices with a 0.25 mg dose of ICG versus ICG image scores for all three surgeons. The median vascularity index for ICG image scores of 0 and 2 differed significantly for all the surgeons (*p* < 0.001). For surgeons 1 and 3, the median vascularity indices for image scores of 0 and 1 also were statistically significant (*p* <$$0.002)$$. This trend was not present with surgeon 2 (*p* =$$0.14$$). No statistically significant difference was found when the median vascularity indices for the image scores of 1 and 2 were evaluated.

**Fig. 3:**
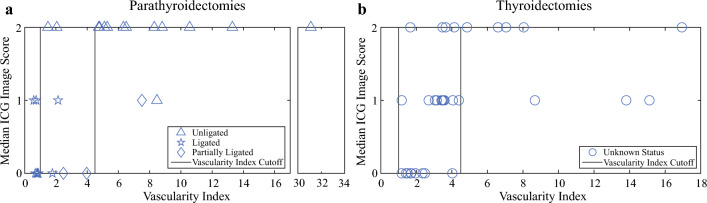
The vascularity indices and median ICG image scores for (a) parathyroidectomies and (b) thyroidectomies. All 59 parathyroid glands were from patients who received a single 0.25 mg dose of ICG. Lines illustrate cutoff points for the vascularity indices. Glands to the left of the line at 1.0 are considered devascularized, glands between the two lines are considered poorly perfused, and glands to the right of the line at 4.5 are well vascularized

Figure 3 shows the median image scores as a function of the vascularity indices in parathyroidectomies and thyroidectomies, respectively. The status of ligation versus no ligation in parathyroidectomy cases was treated as the gold standard for deriving the vascularity index cutoffs between devascularized and well-perfused glands. These cases present a clearer correlation between the vascularity index and the ICG image score, as shown in Fig. 3a. Most glands ligated during the procedures (*n* = 8) had a vascularity index of less than 1.0. All but three of the unligated glands (*n* = 16) had a vascularity index of 4.5 or greater. Of the three misclassified glands, two glands had a high baseline (pre-ICG) fluorescence, minimizing the increase in fluorescence due to ICG. The third gland had a lower post-ICG fluorescence than the other glands in the same patient but received a median image score of 2. Three glands were determined to be insufficiently ligated by the attending surgeon intraoperatively. Two of these glands had a vascularity index between 2.5 and 4.0, whereas the third gland had an index greater than 4.5, indicating significant ICG uptake. Cutoffs for the vascularity index, represented with vertical solid lines at 1.0 and 4.5, were chosen to optimize the agreement between the probe and known status of glands during parathyroidectomy cases, accounting for these variations.

These vascularity index cutoffs then were applied to thyroidectomy cases to estimate the agreement on vascularity between the image scores and the probe-measured fluorescence. Under this framework, no glands were identified as devascularized by the probe. Eight glands were identified as well-vascularized, with a vascularity index of 4.5 or greater. Of these, three glands received a median image score of 1 from the three surgeons. The 24 glands identified as poorly vascularized with a vascularity index between 1.0 and 4.5 had the most amount of disagreement in their assigned image scores, with nine receiving an image score of 0 and four receiving an image score of 2.

## Discussion

This report presents the application of probe-based fluorescence detection in conjunction with low-dose ICG for vascularity assessment of PGs. We show that an ICG dose of 0.25 mg produces a detectable signal with a probe-based system that can be correlated with parathyroid gland perfusion. By focusing on parathyroidectomy cases in which perfusion status was known, vascularity indices were identified that delineated glands with nonexistent, compromised, or excellent blood supply. A vascularity index lower than 1.0 correctly distinguished glands that were completely ligated, with two exceptions, whereas a vascularity index greater than 4.5 indicated an unligated viable gland. The results of this study demonstrate that the use of a probe-based fluorescence detection system enables repeatable objective determination of a PG’s blood supply when low-dose ICG is injected.

To date, no gold standard method exists that can accurately and objectively assess PG viability. The most prevalent technique is visual inspection. Beyond visual inspection, ICG angiography is currently the dominant method used by surgeons for determining PG gland perfusion. With this method, several milligrams of ICG are injected into the patient, and an imaging system is used to detect the ICG via fluorescence as it tracks the blood supply reaching PGs. These fluorescence images are then evaluated with qualitative metrics such as perceived intensity and heterogeneity of ICG uptake to determine gland viability.^20,22,28,35^

Vidal Fortuny et al.^22^ showed that despite consistent use, ICG angiography is limited in its ability to predict postoperative hypoparathyroidism and proposed the use of a qualitative scoring system (0–2) in an effort to standardize its use. However, recent work including the results presented in this report demonstrates the subjective nature of this 0 to 2 scoring system, especially when a poorly perfused gland is scored, which hinders the performance of ICG angiography. For example, Noltes et al.^36^ extracted quantitative imaging information such as the ingress and egress slopes of ICG flow in the PGs and showed that the accuracy of surgeons predicting PG function was 60%, further supporting a need to move away from visually interpreted ICG angiography. Our study corroborates that this disagreement is likely attributable to inter- and intra-rater variability in scoring the ICG images because the vascularity index showed that a gland can undergo a significant increase in fluorescence while still receiving an image score of 0 or 1.

The variability in image scores may be attributable to various factors such as camera position, camera settings such as gain that may have been changed during the operation, ambient light, and the like, leading to subjective scoring. Furthermore, small incisions, large cameras, illumination rings, fluorescent sterile draping, and the need to divert attention from the surgical field to view the image all contribute to additional variations in ICG imaging and scoring. Several groups have proposed alternative strategies to address these shortcomings in ICG imaging. Noltes et al.^36^ and van de Berg et al.^37^ used fixed imaging distances and quantitative pixel metrics to generate well-defined and repeatable measurements for interpretation. McEntee et al.^38^ used machine-learning to determine the viability of glands with corrections for camera movements to normalize images over time. In this study, camera focal lengths and imaging settings were fixed to mitigate the artifacts mentioned earlier, and median image scores were used to mitigate the influence of individual surgeons, effectively using a “majority rules” metric.

In the current study, we demonstrated another approach to evaluating PG viability more reliably with ICG by using a device that can directly measure the intensity of the emitted ICG fluorescence. By setting clear cutoffs between ligated and untouched glands in parathyroidectomies, probe-based fluorescence can account for variability in gland perfusion. For example, two glands were given image scores of 0 by the surgeons despite being partially ligated. These glands, however, had a vascularity index greater than 1.0. Despite the inaccuracy of the image score, their vascularity indices identified them as poorly vascularized, demonstrating the accuracy of probe-based fluorescence detection. Two ligated glands also demonstrated ICG uptake with a vascularity index of approxomately 2.0, indicating that the glands may in fact have not been fully ligated. This same disagreement arose for glands in thyroidectomies. The discrepancy can likely be attributed to the fact that the gland is evaluated relative to its surroundings in the ICG images but relative to the pre-ICG fluorescence in the vascularity index. Further validation of these cutoffs are necessary, but this study provides a step toward the eventual goal of providing surgeons with quantitative feedback on the likelihood that a gland needs autotransplantation, particularly in the case of a poorly perfused gland.

Despite the lack of gold standard for assessing PG viability in thyroidectomies, the findings from these cases provide information on when ICG and the vascularity index are most useful. No glands from thyroidectomies were devascularized according to the vascularity index, and no total thyroidectomy patients experienced hypocalcemia. This fact is in line with research showing that high-volume surgeons have lower rates of postoperative hypocalcemia. However, difficult cases such as those involving central neck dissection have been shown to have an increased rate of hypoparathyroidism. During these more invasive cases, it may prove useful for surgeons to use the vascularity index to assess parathyroid gland viability. Because ICG injection is delayed until the end of the procedure when the vascularity index is used, surgeons can decide at the end whether glands may have been damaged and whether further verification is desired. Similarly, this method can be used in subtotal parathyroidectomy cases to give surgeons the ability to ensure that the remnant gland has an adequate blood supply.

However, this technique has limitations. Glands with a high baseline autofluorescence will show a smaller percentage increase after ICG injection, effectively reducing the vascularity index. Additional studies must be performed to identify a more accurate metric in cases with a gland “too bright” for accurate assessment, as was the case for two glands. The inverse also could prove problematic when a dimly fluorescent area of the gland is used for the baseline, resulting in a greater than normal post-ICG fluorescence change. To account for this, the entire gland should be interrogated before ICG administration to ensure that the brightest region is measured for the baseline.

Overall, probe-based fluorescence detection for objective scoring offers several benefits. By using a probe-based system, the administered ICG dose to evaluate PGs can be significantly reduced (0.25 mg).^22–29^ This reduction in dosage may encourage broader implementation of quantitative ICG angiography for PG viability. Strong fluorescence signals with this dosage also indicate potential to further lower the dosage such that the ICG could be cleared by the liver during the procedure. This could enable ICG administration when one side is finished during a total thyroidectomy while still allowing NIRAF to be used later on the contralateral side.

The probe-based technique is fast, requiring only 4 min for completion after the thyroid has been fully removed. This time, compared with the instantaneous nature of imaging, is required for the small ICG bolus to become evenly distributed throughout the blood before measurement. This approach also is more forgiving. A delay due to losing the gland’s location does not dramatically affect the change in fluorescence when a probe is used. This is in contrast with imaging, in which delays in surgery can result in ICG signal washout with the background signal, making analysis more difficult and necessitating an additional dose of ICG. Using a probe to measure ICG in the PGs also mitigates several issues associated with imaging, such as interference from room lights and the need for vision accommodation by the surgeon. Because the interrogation region is more localized, ICG and blood leaking into the surgical site, causing background noise, are less problematic. The probe is intended to be in contact with the tissue being analyzed, removing the variability of fluorescence detection due to distance and angle in ICG imaging systems.

The use of low-dose ICG with probe detection is a new technique for assessing the blood supply of parathyroid glands. The quantification of fluorescence increase, and therefore the blood supply, enables a quantitative evaluation of the gland’s viability. Prospective studies that implement this approach are necessary before current clinical practice is changed, such as performing autotransplantation when a vascularity index is less than 1.0. The current results from this study provide surgeons with a way to confirm their patient’s chance of postoperative hypoparathyroidism. Additionally, incorporation of this approach into the clinical workflow is flexible, making the method available to surgeons as needed in surgery.

## Conclusions

This study demonstrated a new probe-based fluorescence detection technique together with a small dose of ICG to assess the vascularity of the PGs during thyroidectomies and parathyroidectomies. Use of this approach allows for quantitative evaluation of the blood supply while achieving a 95% reduction of ICG from the standard dosage of ≥ 5 mg.^15,20,29,39^ For surgeons already using NIRAF probes for PG identification, this technique eliminates the need for further instrumentation. Future study is required to validate cutoffs between vascularized and devascularized PGs and to determine the range of PG autofluorescence that works with this technique. However, this is a promising first step toward simultaneous use of probe-based NIRAF and ICG angiography for objective quantification of PG vascularity.

## Supplementary Information

Below is the link to the electronic supplementary material.

Supplementary file1 (DOCX 68 KB)

## Data Availability

The data generated and analyzed in this study are available from the corresponding author, AMJ, upon request.
