# Supplementary material for: Vascularity Assessment of Parathyroid Glands Using Low-Dose ICG and Probe-Based Fluorescence Detection
Source: Ann Surg Oncol. 2026 Feb 20;33(7):5958–65. doi: 10.1245/s10434-026-19250-8 (PMC13242397; doi:10.1245/s10434-026-19250-8)
Supplement: Supplementary file 1 — Supplementary file1 (DOCX 68 KB) [file 10434_2026_19250_MOESM1_ESM.docx]

This document provides supplementary information to “Vascularity Assessment of Parathyroid Glands Using Low-Dose ICG and Probe-Based Fluorescence Detection”

**1. Time Course of Intervention**

The forked nature of this study with respect to surgeries and fluorescence tools may pose a difficulty for readers in understanding the implementation of the intervention. For this reason, we present Figure S1 as an aid for understanding the progression of the clinical portion of the study.


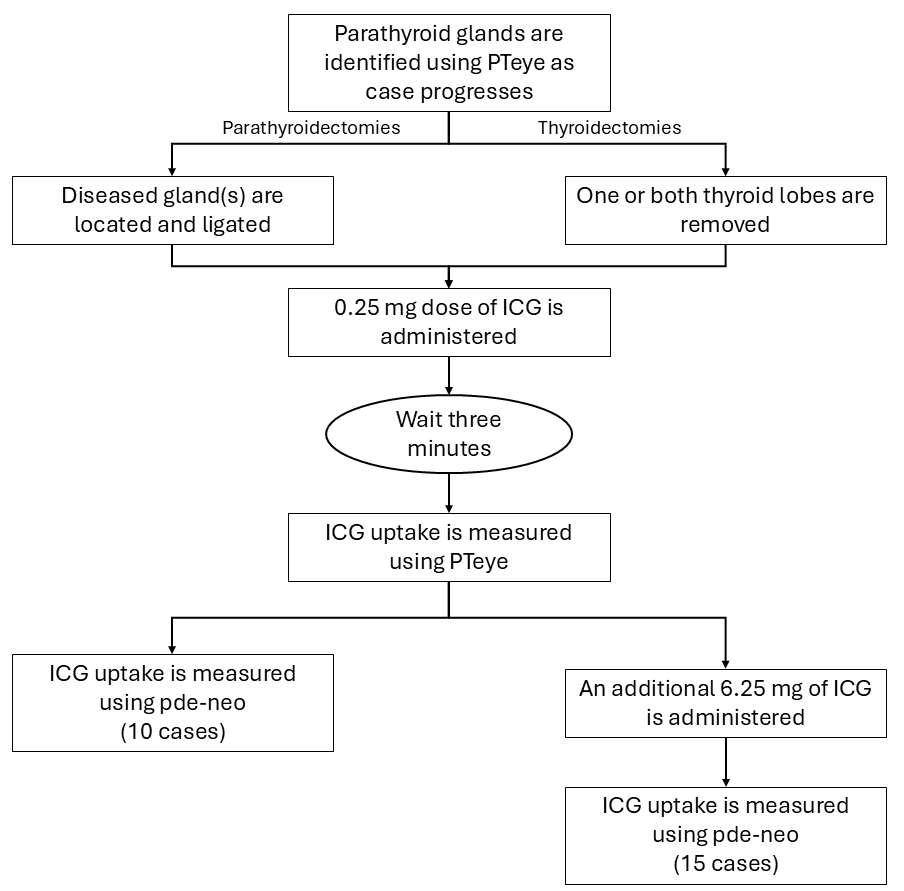


Figure S1. A time course overview of the ICG administration and fluorescence measurement

**2. Parathyroidectomy Sample Size Validation**

Here we detail the statistical justification for the sample size used in parathyroidectomy cases for determining the cutoffs. First, because of the non-normal distribution of the vascularity indices, a standard t-test cannot be used. Thus, a Wilcoxon rank sum test was used. This was done with seven of the eight patients, as Subject 25’s diseased glands were only partially ligated when ICG was administered. Additionally, the outlier gland with a vascularity index of 31.1 was excluded to prevent bias. The test returned a p-value of 0.002, indicating the medians are different between the groups.

We can further check the power of the study to ensure it has a sufficient sample size. Using the means of the unligated and ligated glands, 6.3 and 0.99 respectively, as well as the larger of the two standard deviations (3.4), we find the study is powered at 97.5%.
